# El Niño Southern Oscillation, overseas arrivals and imported chikungunya cases in Australia: A time series analysis

**DOI:** 10.1371/journal.pntd.0007376

**Published:** 2019-05-20

**Authors:** Xiaodong Huang, Wenbiao Hu, Laith Yakob, Gregor J. Devine, Elizabeth A. McGraw, Cassie C. Jansen, Helen M. Faddy, Francesca D. Frentiu

**Affiliations:** 1 School of Biomedical Sciences, Institute of Health and Biomedical Innovation, Queensland University of Technology, Brisbane, Queensland, Australia; 2 School of Public Health and Social Work, Institute of Health and Biomedical Innovation, Queensland University of Technology, Brisbane, Queensland, Australia; 3 Department of Disease Control, London School of Hygiene and Tropical Medicine, London, United Kingdom; 4 Mosquito Control Laboratory, QIMR Berghofer Medical Research Institute, Brisbane, Queensland, Australia; 5 College of Agricultural Sciences, Pennsylvania State University, Pennsylvania, United States of America; 6 Communicable Diseases Branch, Department of Health, Queensland Health, Herston, Queensland, Australia; 7 Research and Development, Australian Red Cross Blood Service, Brisbane, Queensland, Australia; National Center for Atmospheric Research, UNITED STATES

## Abstract

**Background:**

Chikungunya virus (CHIKV) is an emerging mosquito-borne pathogen circulating in tropical and sub-tropical regions. Although autochthonous transmission has not been reported in Australia, there is a potential risk of local CHIKV outbreaks due to the presence of suitable vectors, global trade, frequent international travel and human adaptation to changes in climate.

**Methodology/Principal findings:**

A time series seasonal decomposition method was used to investigate the seasonality and trend of monthly imported CHIKV cases. This pattern was compared with the seasonality and trend of monthly overseas arrivals. A wavelet coherence analysis was applied to examine the transient relationships between monthly imported CHIKV cases and southern oscillation index (SOI) in time-frequency space. We found that the number and geographical distribution of countries of acquisition for CHIKV in travellers to Australia has increased in recent years. The number of monthly imported CHIKV cases displayed an unstable increased trend compared with a stable linear increased trend in monthly overseas arrivals. Both imported CHIKV cases and overseas arrivals showed substantial seasonality, with the strongest seasonal effects in each January, followed by each October and July. The wavelet coherence analysis identified four significant transient relationships between monthly imported CHIKV cases and 6-month lagged moving average SOI, in the years 2009–2010, 2012, 2014 and 2015–2016.

**Conclusion/Significance:**

High seasonal peaks of imported CHIKV cases were consistent with the high seasonal peaks of overseas arrivals into Australia. Our analysis also indicates that El Niño Southern Oscillation (ENSO) variation may impact CHIKV epidemics in endemic regions, in turn influencing the pattern of imported cases.

## Author summary

Chikungunya virus (CHIKV) is mosquito-borne virus circulating in tropical and sub-tropical areas of the globe. Infected travellers from CHIKV-affected areas can initiate outbreaks and epidemics in countries where vector mosquitoes are present. Greater understanding of the pattern of imported cases is required to facilitate risk assessment of CHIKV outbreaks. We investigated the temporal pattern of imported CHIKV cases relative to the pattern of overseas arrivals. We also tested whether variability in El Niño Southern Oscillation (ENSO) can predict the import of CHIKV cases in Australia. We found that the number of monthly imported CHIKV cases displayed an unstable increased trend versus the stable linear increased trend observed in monthly overseas arrivals. Both the numbers of imported CHIKV cases and overseas arrivals showed substantial seasonality. High seasonal peaks of imported CHIKV cases were consistent with the high seasonal peaks of overseas arrivals into Australia. We also identified four significant transient relationships between ENSO variability and CHIKV importation. Our results suggest ENSO may impact the occurrence of CHIKV epidemics in endemic regions, in turn influencing the pattern of imported cases.

## Introduction

Chikungunya virus (CHIKV) is a mosquito-borne viral pathogen transmitted by mosquitoes, primarily *Aedes aegypti* and *Aedes albopictus* [1]. There are no licenced vaccines or specific antiviral drug treatments for the disease [2, 3]. CHIKV has circulated almost continuously in Asia since 1950’s, with over 1.9 million cases reported since 2005 from India, Indonesia, Maldives, Myanmar and Thailand combined [4, 5]. In the Americas, there have been approximately 2.4 million suspected and confirmed cases since December 2013 [6]. CHIKV outbreaks have been occurring in the Pacific since 2011 [7]. There has been substantial geographic expansion of CHIKV outbreaks in the past decade, at least in part due to increased number of international travellers, global trade and evolutionary adaptation of CHIKV to new vector species [8–10].

Infected travellers from affected areas can initiate new CHIKV epidemics in countries where vector mosquitoes are present [11]. CHIKV epidemics triggered by imported CHIKV cases have occurred in the United States, France and Italy [12–16]. Human behavioural/social/land use adaptations to large scale changes in climate may also influence geographical distribution of the CHIKV vector mosquitoes *Ae*. *aegypti* and *Ae*. *albopictus* [11, 17–20]. Further, the global potential of CHIKV epidemics could be affected by weather variation because virus transmission, survival and abundance of *Ae*. *aegypti* and *Ae*. *albopictus* are highly dependent on local climatic conditions [8, 21, 22]. Previous studies have indicated that climate change might cause an increase in the frequency of CHIKV outbreaks [22–25].

Although no locally acquired CHIKV cases have been reported to date in Australia, the increasing number of infected travellers from endemic regions has become an important public health consideration for areas where the vectors are established, particularly in North Queensland, Australia where local transmission of dengue viruses occurs periodically [26, 27]. Indeed, imported dengue cases have led to autochthonous dengue outbreaks in North Queensland [18, 28, 29], vectored by *Ae*. *aegypti*. In 2016, *Ae*. *albopictus* was responsible for a dengue outbreak on the islands of Erub and Badu in the Australian Torres Strait [30]. This was the first *Ae*. *albopictus* mediated dengue outbreak in Australian history. *Ae*. *albopictus* is currently only found on these islands but has the potential to extend its range across mainland Australia [31]. Since the National Notifiable Diseases Surveillance System [32] started reporting chikungunya virus infection in 2008, imported cases to Australia have been recorded [27, 33]. Outbreaks of vector-borne and particularly *Aedes*-borne diseases have been related to the behaviours of El Niño–Southern Oscillation (ENSO) [34–36]. Previous studies have suggested that the occurrence of CHIKV outbreaks might be associated with El Niño events in India and Indonesia [37, 38]. To facilitate CHIKV risk assessment, it is important to understand the pattern of imported cases into Australia. We aimed to investigate the temporal pattern of imported CHIKV cases relative to the pattern of overseas arrivals, as well as identify whether variability in ENSO can predict the import of CHIKV cases in Australia.

## Methods

### Data collection

CHIKV is a notifiable disease in Australia and suspected cases are laboratory confirmed [32]. Monthly imported CHIKV cases were collected from the National Notifiable Diseases Surveillance System [39] for the period from January 2008 to December 2017. The data can be accessed from the Australian Government Department of Health website (http://www9.health.gov.au/cda/source/cda-index.cfm). Data on yearly imported CHIKV cases by country of acquisition were only available from 2013 to 2017 on the Australia Government Department of Health website (http://www.health.gov.au/internet/main/publishing.nsf/Content/ohp-vectorborne-overseas-acquired.htm). We also obtained the monthly imported CHIKV cases from New South Wales (NSW), Queensland (Qld), South Australia (SA), Tasmania (Tas), Victoria (Vic), Western Australia (WA) and Northern Territory (NT), in order to explore the spatiotemporal patterns of monthly imported CHIKV cases among different states and territories across Australia. The Australian Capital Territory (ACT) did not report cases between January 2008 and December 2017. In Australia, the majority of imported CHIKV cases were acquired from the Pacific region, Southeast Asia and South and Central Asia [40]. Thus, we used data on the monthly number of overseas arrivals from those areas between January 2008 and December 2017. The overseas arrivals included all recorded movements across the Australian international border [41]. The overseas arrivals data are available from Australian Bureau of Statistics website (http://www.abs.gov.au/AUSSTATS/abs@.nsf/DetailsPage/3401.0Mar%202018?OpenDocument).

The Southern Oscillation Index (SOI) is used to measure the intensity of El Niño and La Niña events, which strongly affect changes in temperature and precipitation [42, 43]. Thus, we used the global climatic factor of SOI to explore the potential impact of ENSO events on the dynamics of imported CHIKV cases in Australia. The monthly SOI is available from the Australian Government Bureau of Meteorology website (www.bom.gov.au/climate/current/soihtm1.shtml).

### Statistical analysis

We used a time series seasonal decomposition analysis to detect the seasonality and the trends for monthly imported CHIKV cases and monthly overseas arrivals. The model is given by: *Y*_*t*_ *= T*_*t*_ *+ S*_*t*_ *+ E*_*t*_, where *Y*_*t*_ denotes the original times series for the monthly imported cases and the monthly overseas arrivals; *T*_*t*_, *S*_*t*_ and *E*_*t*_ denote the trend and cycle component, the seasonal component and the error component, respectively. The seasonal component can capture the seasonal pattern of the data, a pattern that will likely repeat every year. Analyses were undertaken using IBM SPSS Statistics 25.

Wavelet analysis is one of the most efficient approaches for non-stationary time series data. The method can identify the periodical variability by decomposition of a time series into the time-frequency space [44, 45] and can quantify the temporal evolution of imported CHIKV cases with different periodic components. The Morlet wavelet *φ*(*τ*) was used to construct the continuous wavelet transform *W*_*x*_(*s*,*δ*) and *W*_*y*_(*s*,*δ*) for the monthly imported CHIKV cases time series *x*(*t*) and the monthly overseas arrivals time series *y*(*t*) respectively. A continuous wavelet transform analysis was used to explore localized intermittent periodicities in each time series *x*(*t*) and *y*(*t*). The Morlet wavelet *φ*(*τ*) and the continuous wavelet transform *W*(*s*,*δ*) were given by [44, 45]

$$\varphi (\tau )=\pi^{-1/4}e^{i\omega \tau }e^{-\frac{1}{2}}\tau^{2}$$

$$W(s,\delta )=\frac{1}{\sqrt{s}}\int_{-\infty }^{\infty }x(t)\varphi^{*}(\frac{t-\delta }{s})dt$$

where *τ* is dimensionless time; *ω* is nondimensional frequency; *s* represents a wavelet scale; δ is the localized time index. The (*) shows the operation of complex conjugate. A cross wavelet transform *W*_*xy*_(*s*,*δ*) was described as $W_{xy}(s,\delta )=W_{x}{(s,\delta )W}_{y}^{*}(s,\delta )$, which determines the regions with high common power between *x*(*t*) and *y*(*t*). Wavelet coherence analysis can determine significant coherence with a consistent phase relationship between *x*(*t*) and *y*(*t*) in the time and frequency domain. The wavelet coherence *R*_*xy*_(*s*,*δ*) is defined as:

$$R_{xy}^{2}(s,\delta )=\frac{{\left|S(\frac{1}{s}W_{xy}(s,\delta ))\right|}^{2}}{S(\frac{1}{s}{\left|W_{x}(s,\delta )\right|}^{2})*S(\frac{1}{s}{\left|W_{y}(s,\delta )\right|}^{2})}$$

Here, *S* is a smoothing operator. Following established methods, the number of monthly imported CHIKV cases were converted into a normal distribution by a logarithmic transformation [45]. The 5% significance level was determined using Monte Carlo methods (1000 Monte Carlo randomizations). Wavelet analysis was conducted using the package “biwavelet” in R [46]. The squared wavelet coherence shows as a “heat map”. The phase (lead-lag) relationships between *x*(*t*) and *y*(*t*) are presented by arrows pointing in the heat map. There is a positive (negative) relationship between *x*(*t*) and *y*(*t*) when the arrows point to right (left). The arrows pointing up (down) mean that *x*(*t*) lags (leads) *y*(*t*) by π/2.

In general, SOI fluctuates monthly, while the development of an El Niño or a La Niña event requires negative or positive SOI values for a period of several months [47]. Before starting the wavelet analysis, we examined the correlations between monthly imported CHIKV cases and 2–6 month lagged moving averages of SOI, respectively. We performed linear regression models using SPSS software to examine the relationships between monthly imported CHIKV cases and 2–6 month lagged moving average. The 6-month lagged moving average SOI displayed the highest R^2^ value for the linear regression model and the highest value of correlation coefficient. Thus, the 6-month lagged moving average SOI was used in the wavelet coherence analysis.

## Results

### Descriptive analysis

**Fig 1:**
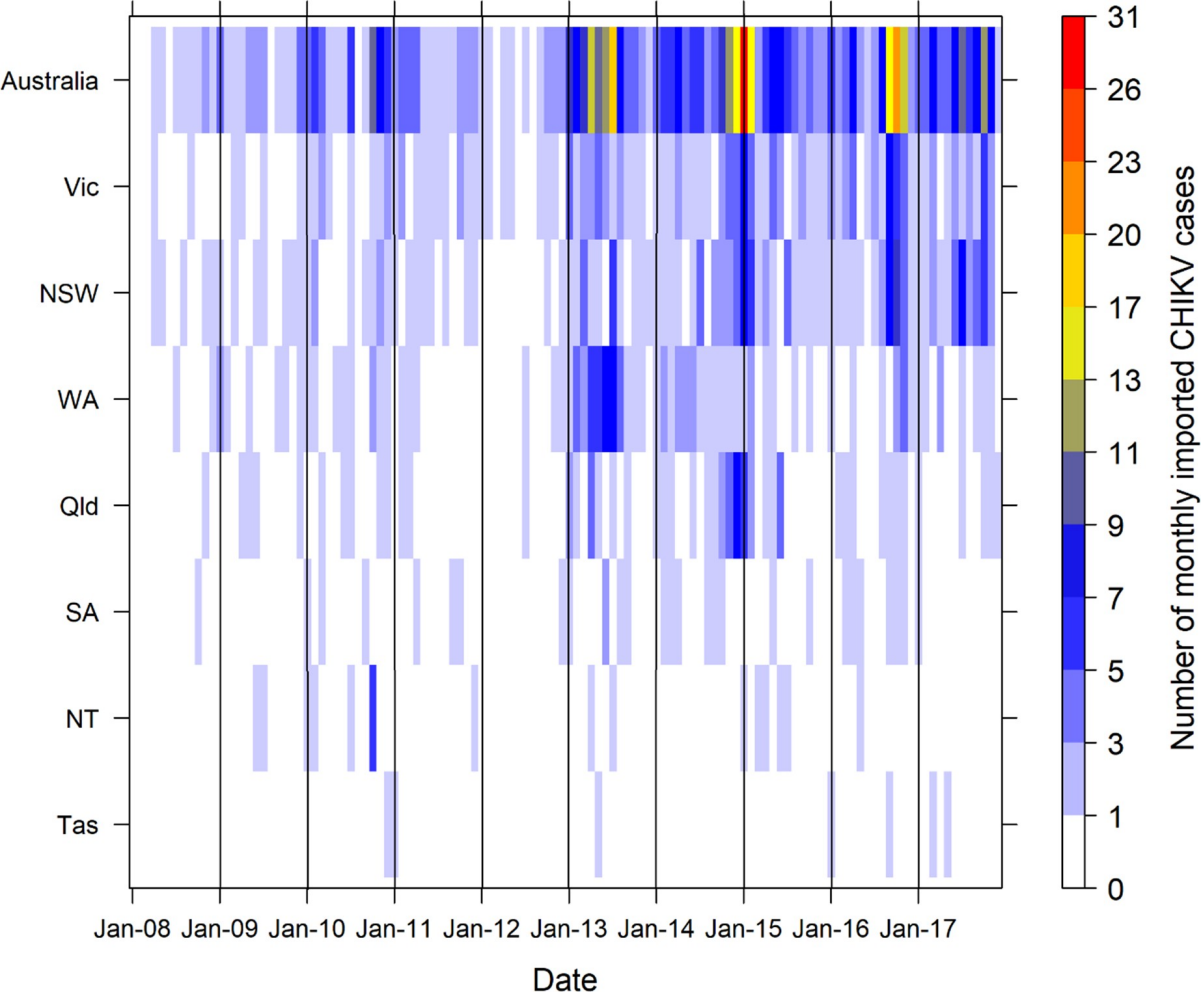
Heat map of the monthly imported CHIKV cases by the states, from January 2008 to December 2017 in Australia.

**Fig 2:**
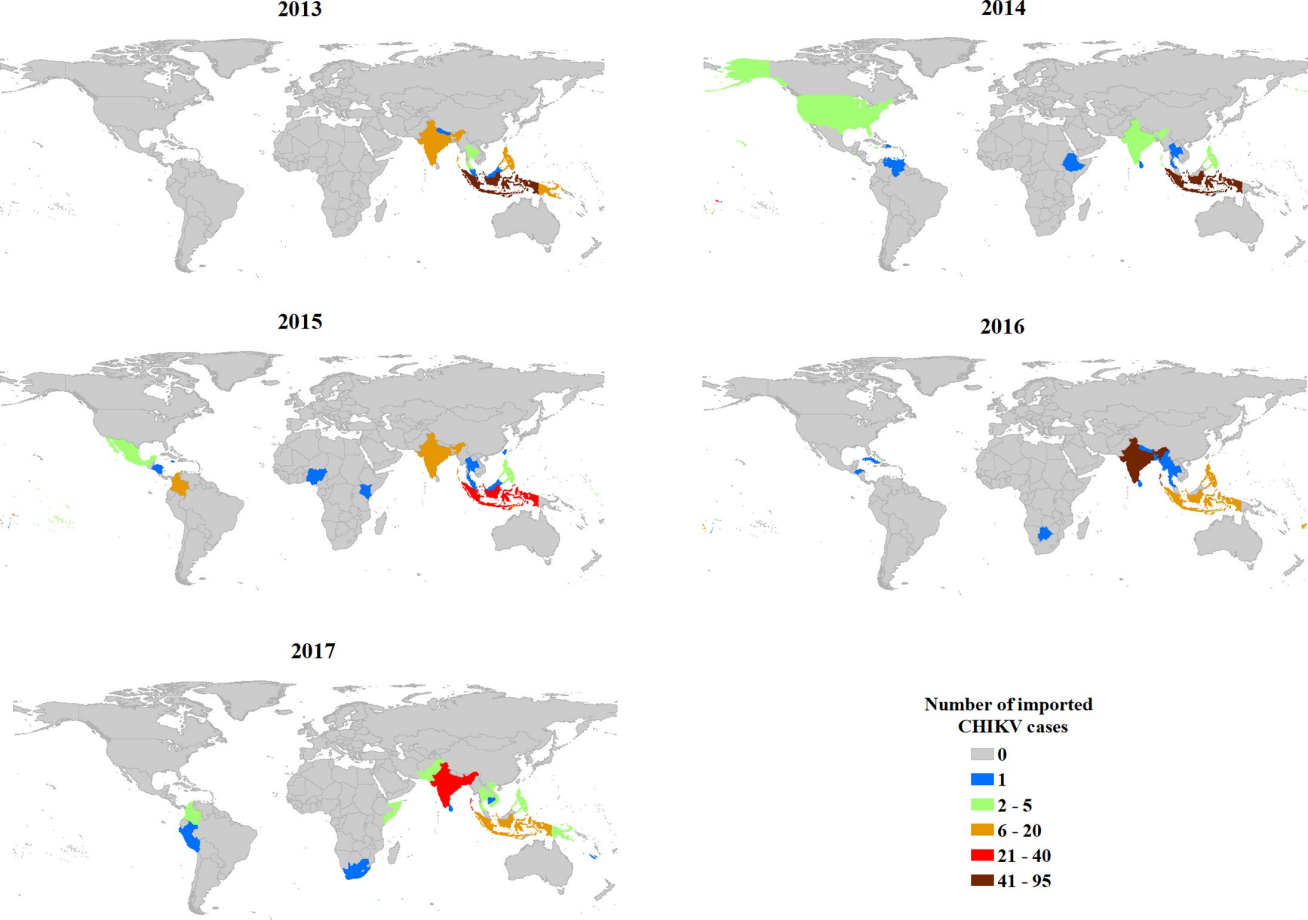
Spatiotemporal variability of annual imported CHIKV cases by countries, from 2013 to 2017 in Australia. The confirmed number of annual CHIKV cases by the countries were presented in S1 Table. Map was made using ArcMap 10.6.

There were 730 imported CHIKV cases from 2008 to 2017 in Australia. The largest number of monthly imported CHIKV cases was 31 cases in January 2015. The largest number of annual imported CHIKV cases occurred in 2013 (134 cases), followed by 2016 (114 cases), 2015 (111 cases) and 2014 (110 cases). The mean of monthly imported CHIKV cases was 6.08 (range: 0–31) during the study period across Australia and varied between states (Table 1). NSW and VIC had the highest means of monthly imported CHIKV cases when compared with the other states (Fig 1). These states also have the largest populations. The frequency of the months with imported CHIKV cases per year has increased since 2013 in NSW (10–11 months), VIC (10–11 months), QLD (5–9 months) and WA (6–12 months) states (Fig 1). Excluding 13 cases with unknown or unclear provenance, the five countries most frequently reported as location of CHIKV acquisition from 2013 to 2017 were Indonesia (33.27% of imported cases), India (20.25%), Samoa (8.27%), Bangladesh (6.34%) and Philippines (4.05%) (Fig 2). There were eight countries of acquisition in 2013, including Indonesia (95 cases), Papua New Guinea (15 cases), India (11 cases), Philippines (6 cases), Thailand (3 cases), Singapore (2 cases), Malaysia (1 case) and Nepal (1 case). However, after 2013, the number of source countries of imported CHIKV cases substantially increased and ranged from 15 to 24 countries per year. Since 2014, the geographical distribution of countries of acquisition has expanded from Southeast Asia, South and Central Asia and Southwest Pacific region to include Eastern Africa, Western Africa, Southern Africa, North America, Central America, South America and the south Pacific regions (Fig 2).

**Table 1:** Summary statistics of number of monthly imported CHIKV cases from January 2008 to December 2017 across states and territories of Australia.

	Mean	SD	Minimum	Maximum
Australia	6.08	5.96	0	31
NSW	1.78	2.39	0	12
NT	0.20	0.75	0	7
Qld	0.70	1.42	0	9
SA	0.28	0.57	0	3
Tas	0.08	0.34	0	2
Vic	1.83	1.99	0	11
WA	1.23	1.91	0	11

**Fig 3:**
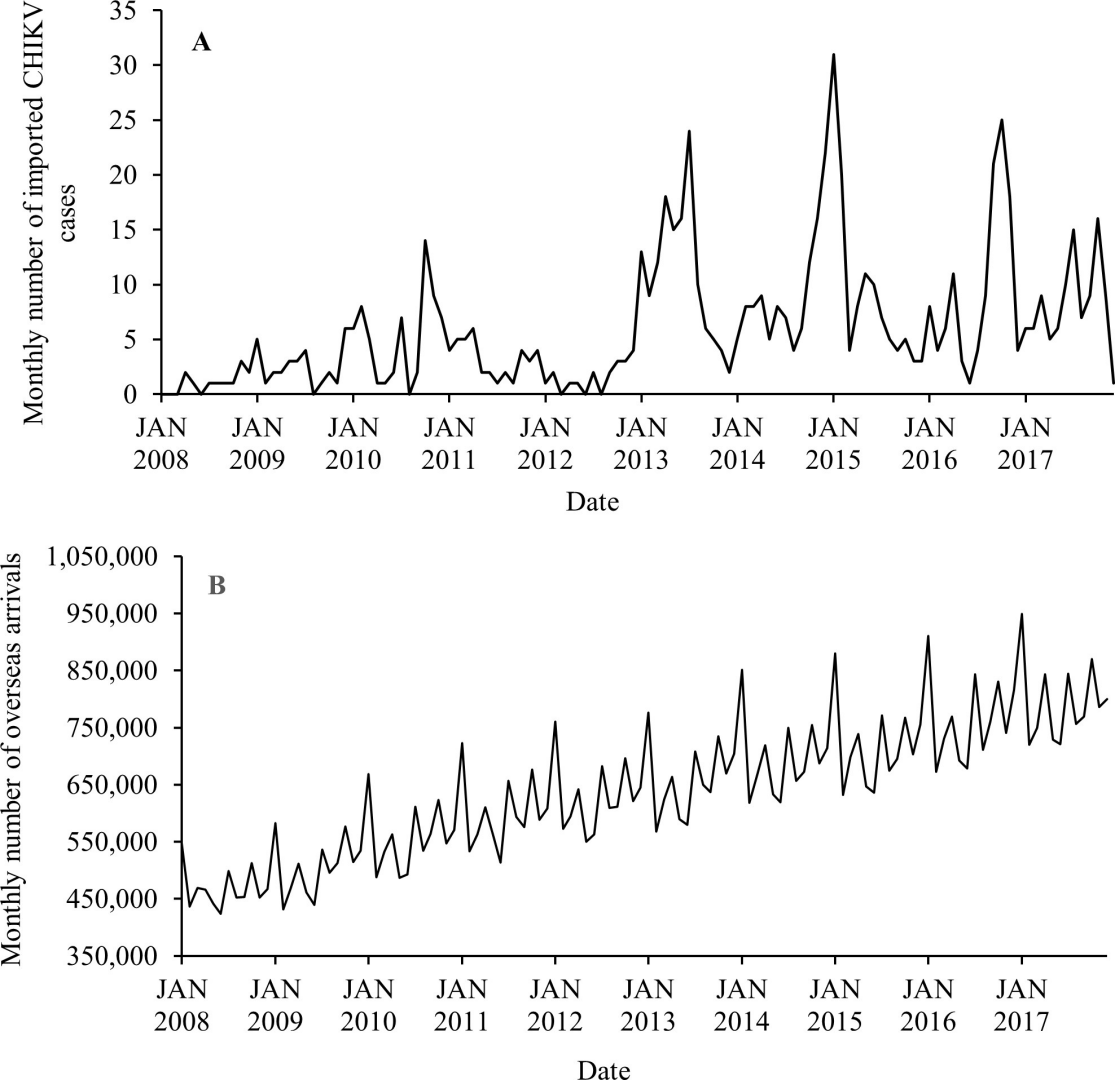
Temporal variation in the numbers of monthly imported CHIKV cases (A) and the monthly overseas arrivals from the Pacific region, Southeast Asia, South and Central Asia (B), from January 2008 to December 2017.

Temporal variabilities of the monthly imported CHIKV cases and the monthly overseas arrivals from the Pacific region, Southeast Asia, South and Central Asia in Australia from January 2008 to December 2017 are displayed in Fig 3. An increase in the number of monthly imported CHIKV cases has occurred from the beginning of 2013 (Fig 3A). Although increased trends for both monthly imported CHIKV cases and monthly overseas arrivals were observed over the study period (Fig 3B), the average of monthly imported CHIKV cases increased by 605.3% from 2012 to 2013, while the average of monthly overseas arrivals only increased by 3.6% from 2012 to 2013. The increase in overseas arrivals was more steady and regular over the whole study period.

### Seasonality and trend

**Fig 4:**
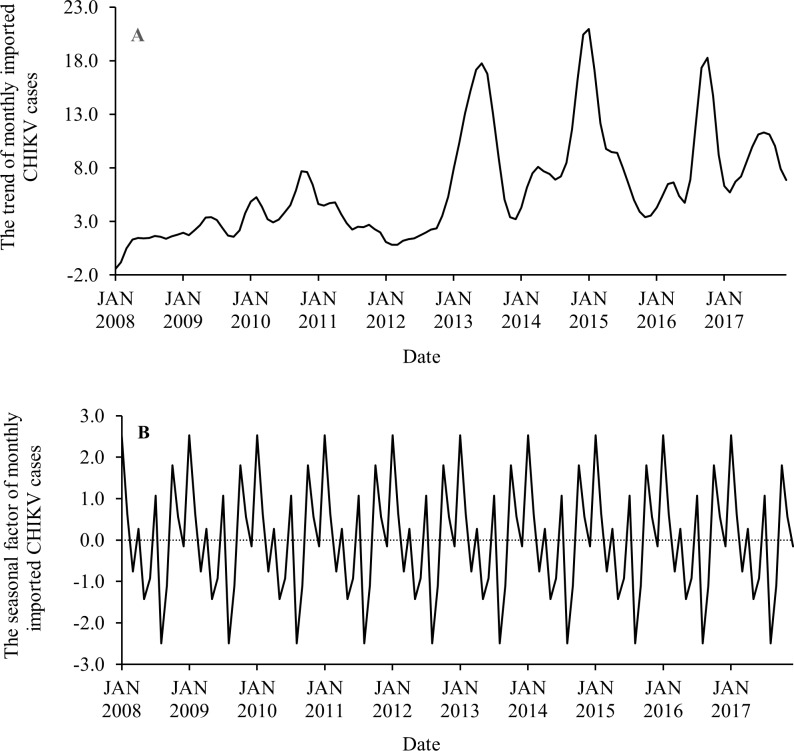
Seasonal decomposition analysis showing the trend (A) and seasonal factor (B) of monthly imported CHIKV cases, from January 2008 to December 2017.

Seasonal decomposition analysis showed that the monthly number of imported CHIKV cases displayed an increased trend from 2008 to 2015, with a slight decreased trend from 2016 to 2017 (Fig 4A). Seasonal decomposition analysis also indicated that there was significant seasonality for monthly imported CHIKV cases (Fig 4B). For the monthly imported CHIKV cases, the strongest positive seasonal effects were found in each January, followed by October, July, February, November and April. The strongest negative seasonal effects appeared in each August, followed by May, September, June, March and December.

**Fig 5:**
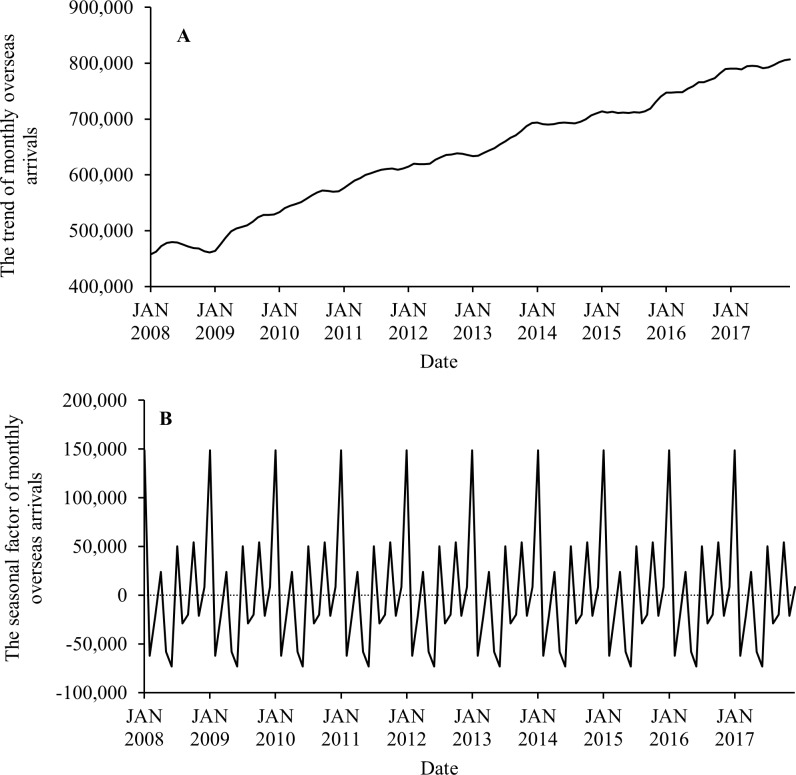
Seasonal decomposition analysis showing the trend (A) and seasonal factor (B) of monthly overseas arrivals, from January 2008 to December 2017.

In contrast to imported CHIKV cases, the monthly number of overseas arrivals exhibited a linear increased trend during the study period (Fig 5A). There was also a strong component of seasonality. For the monthly overseas arrivals, the strongest positive seasonal effects occurred in each January, followed by October, July, April and December, while the strongest negative seasonal effects were observed in each June, followed by February, May, August, November, March and September (Fig 5B).

### Wavelet coherence analysis

**Fig 6:**
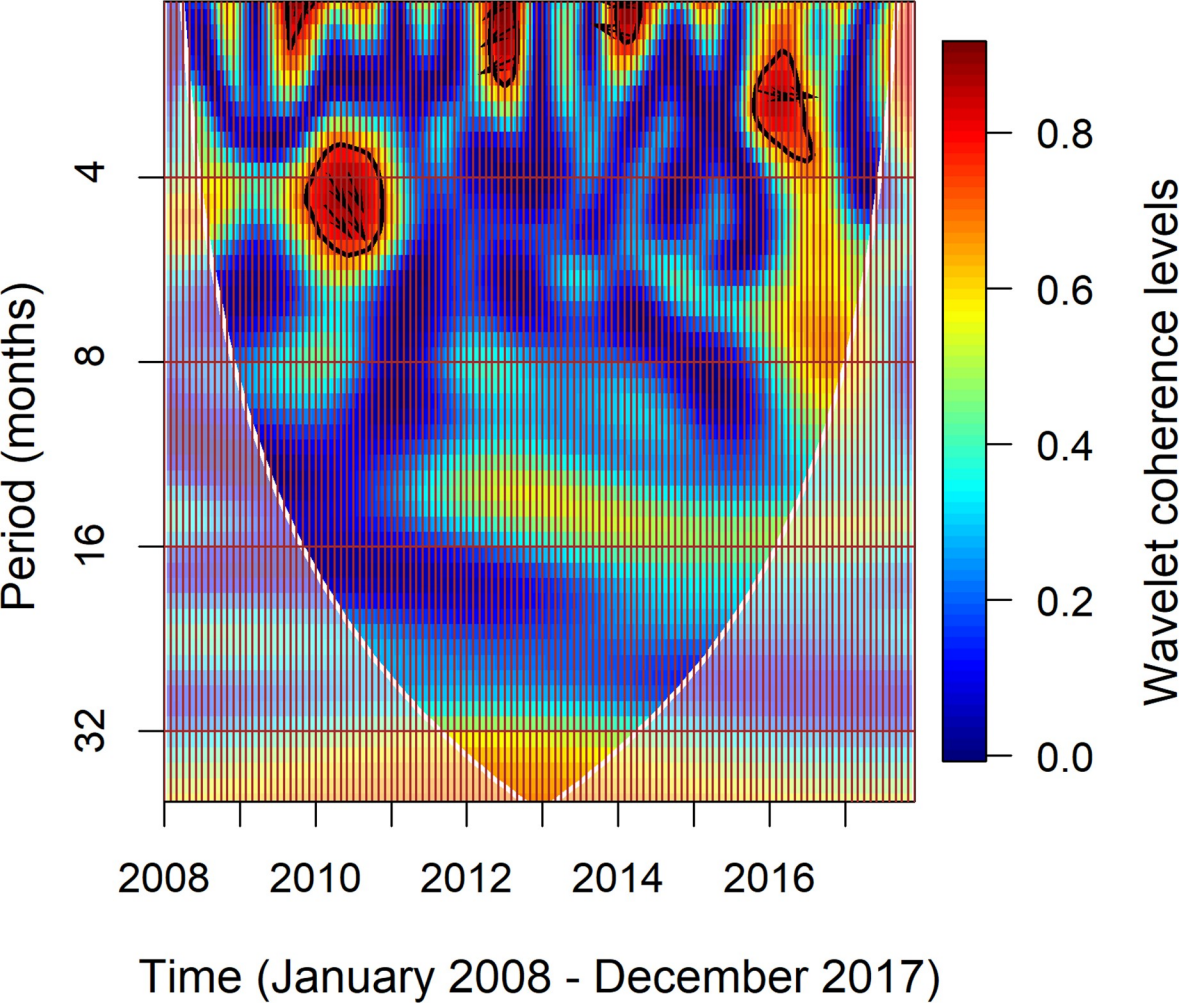
Squared wavelet coherence between monthly imported CHIKV cases and 6-month lagged moving average SOI. The vertical axis shows the frequency of wavelet coherence. The brown vertical grid lines indicate month of the year. The area below the white dashed lines is the ‘cone of influence’, where edge effects restrict the ability to explain the results (shown as a lighter shade). The dark red colour represents the higher correlation between them. The thick black contour designates the 5% statistical significance levels against red noise. The 5% significance levels determined using Monte Carlo methods (1000 Monte Carlo randomizations). Arrow denotes a specific phase relationship between monthly imported CHIKV cases and 6-month lagged moving average SOI.

The wavelet coherence analysis captured some significant transient relationships between monthly imported CHIKV cases and the 6-month lagged moving average SOI in the time frequency space. The heat map (Fig 6) shows in which periods there were significant correlations between the two time series at a given point in time. The monthly imported number of CHIKV cases was significantly positively associated with the 6-month lagged moving average SOI with a slight lead phase, with a period of ~ 3.5–6 months from December 2009 to October 2010 (Fig 6). There were significant negative relationships between the monthly imported CHIKV cases and the 6-month lagged moving average SOI, with a period of ~ 2–3 months during May to August 2012 with a slight lag phase, and with a period of ~ 2–2.5 months from January to April 2014 with a slight lead phase. The numbers of monthly imported CHIKV cases were significantly and positively related to the 6-month lagged moving average SOI, with a period of ~ 2.6–3.8 months from November 2015 to June 2016.

## Discussion

An increased number of countries of acquisition for CHIKV cases imported into Australia was observed during the study period, supporting previous reports of global expansion of CHIKV endemicity [1, 48, 49]. The Pacific region and Southeast/South/Central Asia were the most frequent sources of imported CHIKV cases in Australia, particularly Indonesia and India. From 2014, the geographic range of countries of acquisition expanded to include United States of America, South America, Caribbean Islands, South Africa, and Eastern/Western Africa. Although the number of international arrivals displayed a stable linear increasing trend over the study period, the monthly imported CHIKV cases did not follow the same trend and displayed some instability. It is likely that the trend of monthly imported CHIKV cases reflects the most recent dynamics of CHIKV epidemics in the most frequent countries of acquisition and the number of passenger movements from large epidemic countries.

There were also the peaks in overseas importation from specific countries that coincided with large epidemics observed in those regions. For example, there were massive CHIKV outbreaks in the Americas in 2014 [50], along with the first local chikungunya outbreaks in June 2014 in Florida in the continental United States [51] and in the U.S. Virgin Islands [52]. These outbreaks coincided with CHIKV importation into Australia in 2014 from Barbados, Dominican Republic, El Salvador, Grenada, Jamaica, United States of America and Venezuela. A major CHIKV outbreak was reported in Papua New Guinea in 2013 [7], which resulted in the largest number of imported CHIKV cases into Australia during the period of 2013–2017. Samoa and Tonga experienced large CHIKV outbreaks in 2014 which corresponded to the high numbers of imported CHIKV cases into Australia in 2014 [7]. A substantial CHIKV outbreak was reported in India in 2016 [53], which also led to 54.39% of CHIKV cases into Australia originating in India that year. Bangladesh experienced a significant chikungunya outbreak in 2017 [54], with Australia observing the largest number of imported CHIKV cases in 2017 being from Bangladesh. Moreover, it is notable that there was an increase in the frequency and number of imported CHIKV cases into Australia over the study period, suggesting that the potential risk of autochthonous outbreak will increase if there is sufficient vector mosquito density under suitable weather conditions, in regions with competent vectors in Australia.

From the seasonal decomposition analysis, although the seasonal patterns of monthly imported CHIKV cases and monthly overseas arrivals were not fully synchronized, we observed the seasonal effects on both imported CHIKV cases and overseas arrivals to be strongest each January, followed by October and July. It is likely that the summer holiday period might facilitate the number of imported CHIKV cases due to returning international travellers during the peak Australian vacation season. Additionally, the second and third highest seasonal factor values occurred in each October and July, periods that coincide with Australian school and university holidays.

Interestingly, the timing of the lowest seasonal effects exhibited a noticeable difference. For example, the weakest seasonal effects on monthly imported CHIKV cases occurred each August, followed by May and September, while monthly overseas arrivals were at their lowest in each June, followed by February and May. Almost all countries of acquisition identified in our study experience a tropical and subtropical climate and many experience a dry season from June to September (e.g. Fiji, Kiribati, Indonesia, Malaysia, Samoa, Singapore, Papua New Guinea and the Philippines). Most of India experiences very hot summer weather during April to September [55]. Dry seasons and high temperatures may likely decrease vector abundance in source countries by reducing the production and survival rate of vectors, decreasing the likelihood of CHIKV outbreaks [56]. Overall, the results confirmed that temporal variation in imported CHIKV cases might be driven not only by the behaviour of international travellers but also by the dynamics of CHIKV outbreaks and epidemics in the source countries.

To our knowledge, this is the first study to explore the impact of ENSO on CHIKV importation to non-endemic areas. The wavelet analysis revealed significant positive relationships between the monthly imported CHIKV cases and the 6-month lagged moving average SOI during December 2009 to October 2010 and from November 2015 to June 2016. Historically, there were EI Niño events from 2009 to 2010 and from 2015 to 2016 [57]. During 2009 to 2010, the main acquisition countries included Indonesia, India and Malaysia [26]. Large CHIKV outbreaks were reported across India in 2010 [58], and Indonesia in 2009 (83,756 reported cases) and 2010 (53,899 reported cases) [38]. A large number of CHIKV cases were observed in 2010 in Bali, Indonesia, [38], a popular destination for international travellers, particularly Australians. Moreover, Malaysia reported large outbreaks during 2008 to 2010 [59], which is also a popular destination for international travellers.

The relationship between arboviral transmission and temperature and rainfall is complex and regionally inconsistent [60]. However, up to a threshold, temperature generally has a positive relationship with *Aedes* spp. abundance [56]. Similarly, moderate rainfall is generally found to favour higher vector abundances [30]. El Niño events are typically related to warm and dry weather conditions [57]. Moderate to high temperatures could facilitate increased CHIKV transmission because increasing temperature leads to a higher biting rate and shorter extrinsic incubation period for vector mosquitoes [34]. Conversely, extremely high temperature could increase the mortality rate of mosquitoes [61]. Increased SOI could lead to a decrease in the intensity of El Niño conditions during such events [62]. Thus, the positive relationship between the monthly imported CHIKV cases and the 6-month lagged moving average SOI suggested that moderate weather conditions under El Niño episodes might enhance the importation of CHIKV.

Conversely, we also observed significant negative relationships between the number of monthly imported CHIKV cases and the 6-month lagged moving average SOI for the period May-August 2012 and the period January-April 2014. For these periods, an increase in SOI was likely to decrease the number of monthly imported CHIKV cases under neutral ENSO conditions (i.e. absence of La Niña or El Niño). Increased SOI could increase the chance of a La Niña event developing during the neutral ENSO period. La Niña typically leads to cold temperature and heavy rain weather conditions. Heavy rainfall could flush out mosquito breeding sites [63], leading to lowered CHIKV transmission risk. Conversely, neutral ENSO conditions that lead to equable weather conditions with low to moderate rainfall may lead to increased CHIKV transmission risk. It is interesting that in the period from 2012–2014 a series of CHIKV epidemics occurred in the Pacific [7, 64], which led to large numbers of imported CHIKV cases into Australia. Our results suggest that ENSO behaviours could drive local environmental conditions, which lead to changes in the patterns of local CHIKV epidemics in different endemic regions.

### Limitations

Some limitations of this study should be acknowledged. First, the imported CHIKV cases used in this analysis were only from laboratory confirmed individuals and therefore did not include infected persons who were asymptomatic or suffered only mild symptoms and thus did not seek medical advice. Second, the data by countries of acquisition were only publicly available for annual imported cases from 2013 to 2017. Therefore, we could not provide the spatiotemporal distribution of imported CHIKV cases by countries across the whole study period. We were also unable to calculate monthly incidence rates (numbers of imported cases over overseas arrivals) for each particular country over the study period, as the latter set of data were not publicly available. Third, the impact of ENSO could result in varying climate conditions in different parts of the world. Using aggregated data on monthly imported CHIKV cases from all source countries in the study could not fully reveal the associations between ENSO behaviours and imported CHIKV cases. Future studies may address the problem by using the imported CHIKV cases by countries or by a group of countries where they have the same weather conditions when the data are available. Additionally, future studies should investigate whether there are differences in the effects of different ENSO events, such as El Niño and La Niña, on local CHIKV transmission in endemic countries. The overall trend of increased number of CHIKV imports suggests that Australia remains at risk of a CHIKV outbreak. The patterns of importation we observed for Australia are likely to be mirrored in other countries that are non-endemic for CHIKV and have strong travel links with the countries of acquisition identified here. Australian health authorities have acknowledged the increasing risk of local CHIKV outbreaks, particularly in North Queensland, through the development of the Queensland Chikungunya Management Plan in 2014 [65].

### Conclusions

We found that the geographical distribution of countries of acquisition has expanded significantly, with the frequency and number of imported CHIKV cases into Australia increasing in recent years. Although the timing of the highest seasonal peak imported cases was consistent with overseas arrivals, the lowest seasonal peak overseas arrivals did not result in the lowest seasonal peak imported cases in Australia. The present study can provide useful information for understanding the seasonality of CHIKV risk and guide surveillance for health authorities at corresponding times of the year. Messaging might be tailored to particular seasons, importation pathways and epidemics in key countries of acquisition. It is likely that the dynamics of imported cases are related to the behaviours of ENSO. Our results suggest that climate could be a potential important driver of CHIKV epidemics and that ENSO behaviours could be a useful predictor to forecast mosquito-borne disease epidemics in endemic countries.

## Supporting information

S1 Table Confirmed number of annual imported CHIKV cases by countries, from 2013 to 2017, in Australia. (DOCX)
