# Supplementary material for: El Niño Southern Oscillation, overseas arrivals and imported chikungunya cases in Australia: A time series analysis
Source: PLoS Negl Trop Dis. 2019 May 20;13(5):e0007376. doi: 10.1371/journal.pntd.0007376 (PMC6544329; doi:10.1371/journal.pntd.0007376)
Supplement: S1 Table — (DOCX) [file pntd.0007376.s001.docx]

S1 Table. Confirmed number of annual imported CHIKV cases by countries, from 2013 to 2017 in Australia.

| Country | 2013 | 2014 | 2015 | 2016 | 2017 |
| --- | --- | --- | --- | --- | --- |
| American Samoa | 0 | 1 | 0 | 0 | 0 |
| Bangladesh | 0 | 0 | 0 | 1 | 35 |
| Barbados | 0 | 1 | 0 | 0 | 0 |
| Botswana | 0 | 0 | 0 | 1 | 0 |
| Burma | 0 | 0 | 0 | 1 | 0 |
| Cambodia | 0 | 0 | 0 | 0 | 1 |
| Colombia | 0 | 0 | 9 | 0 | 2 |
| Cook Islands | 0 | 0 | 5 | 0 | 0 |
| Cuba | 0 | 0 | 0 | 1 | 0 |
| Dominican Republic | 0 | 1 | 0 | 0 | 0 |
| East Timor | 0 | 0 | 11 | 4 | 0 |
| El Salvador | 0 | 2 | 1 | 0 | 0 |
| Ethiopia | 0 | 1 | 0 | 0 | 0 |
| Fiji | 0 | 0 | 0 | 11 | 0 |
| French Polynesia | 0 | 0 | 3 | 0 | 0 |
| Grenada | 0 | 2 | 0 | 0 | 0 |
| Honduras | 0 | 0 | 1 | 1 | 0 |
| India | 11 | 4 | 12 | 62 | 26 |
| Indonesia | 95 | 45 | 23 | 18 | 8 |
| Jamaica | 0 | 4 | 1 | 0 | 0 |
| Kenya | 0 | 0 | 1 | 0 | 0 |
| Kiribati | 0 | 0 | 5 | 0 | 0 |
| Malaysia | 1 | 0 | 1 | 0 | 0 |
| Mexico | 0 | 0 | 3 | 0 | 0 |
| Nauru | 0 | 0 | 5 | 0 | 0 |
| Nepal | 1 | 0 | 0 | 1 | 0 |
| New Caledonia | 0 | 0 | 0 | 0 | 1 |
| Nicaragua | 0 | 0 | 1 | 0 | 0 |
| Nigeria | 0 | 0 | 1 | 0 | 0 |
| Pakistan | 0 | 0 | 0 | 0 | 2 |
| Papua New Guinea | 15 | 0 | 0 | 0 | 2 |
| Peru | 0 | 0 | 0 | 0 | 1 |
| Philippines | 6 | 3 | 2 | 7 | 5 |
| Samoa | 0 | 25 | 20 | 2 | 0 |
| Singapore | 2 | 1 | 0 | 0 | 0 |
| Somalia | 0 | 0 | 0 | 0 | 4 |
| South Africa | 0 | 0 | 0 | 0 | 1 |
| Sri Lanka | 0 | 1 | 0 | 1 | 1 |
| Taiwan | 0 | 0 | 1 | 0 | 0 |
| Thailand | 3 | 1 | 1 | 1 | 4 |
| Tonga | 0 | 7 | 1 | 1 | 0 |
| Trinidad and Tobago | 0 | 1 | 0 | 0 | 0 |
| United States | 0 | 2 | 0 | 0 | 0 |
| Venezuela | 0 | 1 | 0 | 0 | 0 |
| Vietnam | 0 | 0 | 0 | 0 | 4 |
